# Monitoring of spine curvatures and posture during pregnancy using surface topography – case study and suggestion of method

**DOI:** 10.1186/s13013-016-0099-2

**Published:** 2016-10-17

**Authors:** Jakub Michoński, Katarzyna Walesiak, Anna Pakuła, Wojciech Glinkowski, Robert Sitnik

**Affiliations:** 1 Department of Virtual Reality Techniques, Institute of Micromechanics And Photonics, Boboli 8, Warsaw, 02-525 Poland; 2 Department of Orthopedics and Traumatology of the Locomotor System, Baby Jesus Clinical Hospital, Lindleya 4, Warsaw, 02-005 Poland; 3 Department of Medical Informatics and Telemedicine, Medical University of Warsaw, Banacha 1a, Warsaw, 02-097 Poland

## Abstract

**Background:**

Low back and pelvic pain is one of the most frequently reported disorders in pregnancy, however etiology and pathology of this problem have not been fully determined. The relationship between back pain experienced during pregnancy and posture remains unclear. It is challenging to measure reliably postural and spinal changes at the time of pregnancy, since most imaging studies cannot be used due to the radiation burden. 3D shape measurement, or surface topography (ST), systems designed for posture evaluation could potentially fill this void. A pilot study was conducted to test the potential of monitoring the change of spine curvatures and posture during pregnancy using surface topography. A single case was studied to test the methodology and preliminarily assess the usefulness of the procedure before performing a randomized trial. The apparatus used in this study was metrologically tested and utilized earlier in scoliosis screening.

**Case presentation:**

The subject was measured using a custom-made structured light illumination scanner with accuracy of 0.2 mm. Measurement was taken every 2 weeks, between 17th and 37th week of pregnancy, 11 measurements in total. From the measurement the thoracic kyphosis and lumbar lordosis angles, and vertical balance angle were extracted automatically. Custom-written software was used for analysis. Oswestry Low Back Pain Disability Questionnaire (ODI) was done with every measurement. The values were correctly extracted from the measurement. The results were: 50.9 ± 2.4° for kyphosis angle, 58.1 ± 2.1° for lordosis angle and 4.7 ± 1.7° for vertical balance angle. The registered change was 7.4° in kyphosis angle, 8.4° in lordosis angle and 5.5° in vertical balance angle. The calculated ODI values were between moderate disability and severe disability (22 to 58 %).

**Conclusions:**

This case study presents that surface topography may be suitable for monitoring of spinal curvature and posture change in pregnant women. The ionizing radiation studies are contraindicated during pregnancy. Surface topography data connected with information from pain level questionnaires allows to investigate the connection between changes in posture and back pain.

## Background

Lower back pain during pregnancy is a well-known problem. Ostgaard et al. report that almost 50 % pregnant women suffer from back pain [1]. Gutke et al. find that the frequency of lower back pain in pregnant women can be up to four times higher than in non-pregnant women [2]. Pain during pregnancy can have a significant negative impact on day to day functioning affecting ability to work and sleep [3]. In the prospective study of Kristiansson et al. 30 % women with the highest pain score report great difficulties with normal activities [4]. According to Fast et al. [5] more than one-third of pregnant women suffer from back pain at night, which results in a chronic sleeplessness. What is more, about 30 % of pregnant women reduce their physical activity because of back pain [6]. Pregnancy-related lower back pain can also increase the risk of stress and feelings of low mood anxiety [7]. Despite that low back and pelvic pain is one of the most frequently reported disorders in pregnancy, etiology and pathology of this problem have not been fully determined [8]. Lack of understanding of these problems seems to be due to diagnostic restrictions during pregnancy. It is challenging to measure reliably postural and spinal changes at the time of pregnancy, since most imaging studies cannot be used due to the radiation burden [9]. 3D shape measurement, or surface topography (ST), systems designed for posture evaluation could potentially fill this void.

Pain located in the lumbosacral region and pelvis can be caused by various changes occurring during the pregnancy. Gestational weight and its asymmetrical distribution causes that pregnant women arch their backs to move the center of mass of the upper body backward what increases the load on the facet joints. What is more growth of the uterus causes lengthening of abdominal muscles and may allow lumbar lordosis to increase [10, 11]. Moreover, pregnancy-related lower back pain may occur due to dysfunction of the pubic symphysis, sacroiliac joints or hip joints, hormonal loosening of the pelvic ligaments, or peripheral circulatory disorders [12]. Several risk factors have also been identified including the previous history of lower back pain and low satisfaction of the job [13].

The relationship between back pain experienced during pregnancy and posture remains unclear. Moore at al. performed a study on the postural changes in pregnant women in 1989 [14], however, did not use surface topography to achieve this goal. Karras and Tympandis have used 3D measurements to asses shape variation during pregnancy. Measurements of volume and area of the abdomen, buttocks, breasts and thighs have been made at various stages of pregnancy and after delivery [15, 16]. Bullock et al. found no relationship between changes during pregnancy or spinal posture magnitude and back pain [17]. The aim of this study was to test the potential of monitoring the change of spine curvatures and posture during pregnancy using surface topography.

## Methods

**Fig. 1:**
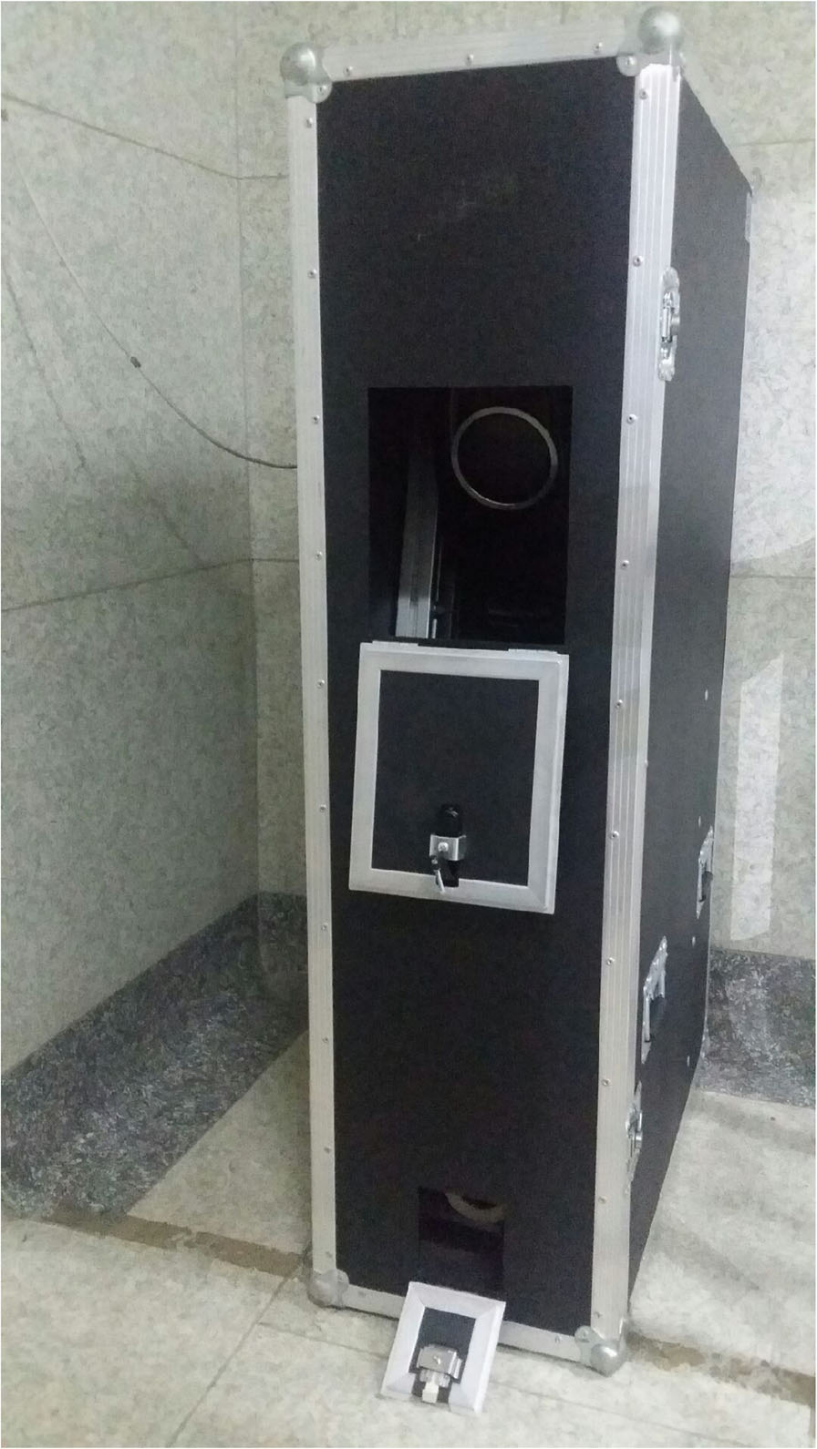
Measurement system used in the study

A single case was investigated to test the methodology and preliminarily assess the usefulness of the procedure before performing a randomized trial. The subject was measured using a custom-made structured light illumination (SLI) scanner with an accuracy of 0.2 mm, built using a DLP projector and an industrial camera (Fig. 1). Duration of the measurement was 0.9 s. Results were produced in the form of a point cloud. The apparatus was metrologically tested and utilized earlier in scoliosis screening [18].

The subject was 34 years old at the beginning of the study, no systemic disorder, no drug use, no previous trauma or surgery of the spine or lower limbs. The measurements were performed during the third full-term pregnancy of this patient. The subject did not have back pain before pregnancies. The patient worked until the end of pregnancy during which the measurements were made. Type of work was identified as 60 % sitting and 40 % standing. The patient has not practiced any sports. Pregnancy lasted 38 weeks and ended by vaginal delivery.

Measurements were taken every 2 weeks, between 17th and 37th week of pregnancy, 11 measurement sessions in total. Each session consisted of three measurements to follow natural changes in posture. Between the measurements, the subject was asked to walk around and then come back to the measurement spot. From the values calculated based on all the measurements, the median value in each session was chosen. Oswestry Low Back Pain Disability Questionnaire (ODI) was done after each session [19].

The plumb line of each measurement was previously aligned using calibration of the measurement system. Additional rotation around the vertical axis was removed by manually locating the posterior superior iliac spines (PSIS) on the point cloud and applying an additional transformation to place them in the frontal plane. From the measurement the thoracic kyphosis and lumbar lordosis angles, and vertical balance were extracted. Each measurement was analyzed separately using a custom-written software based on the FRAMES library.

**Fig. 2:**
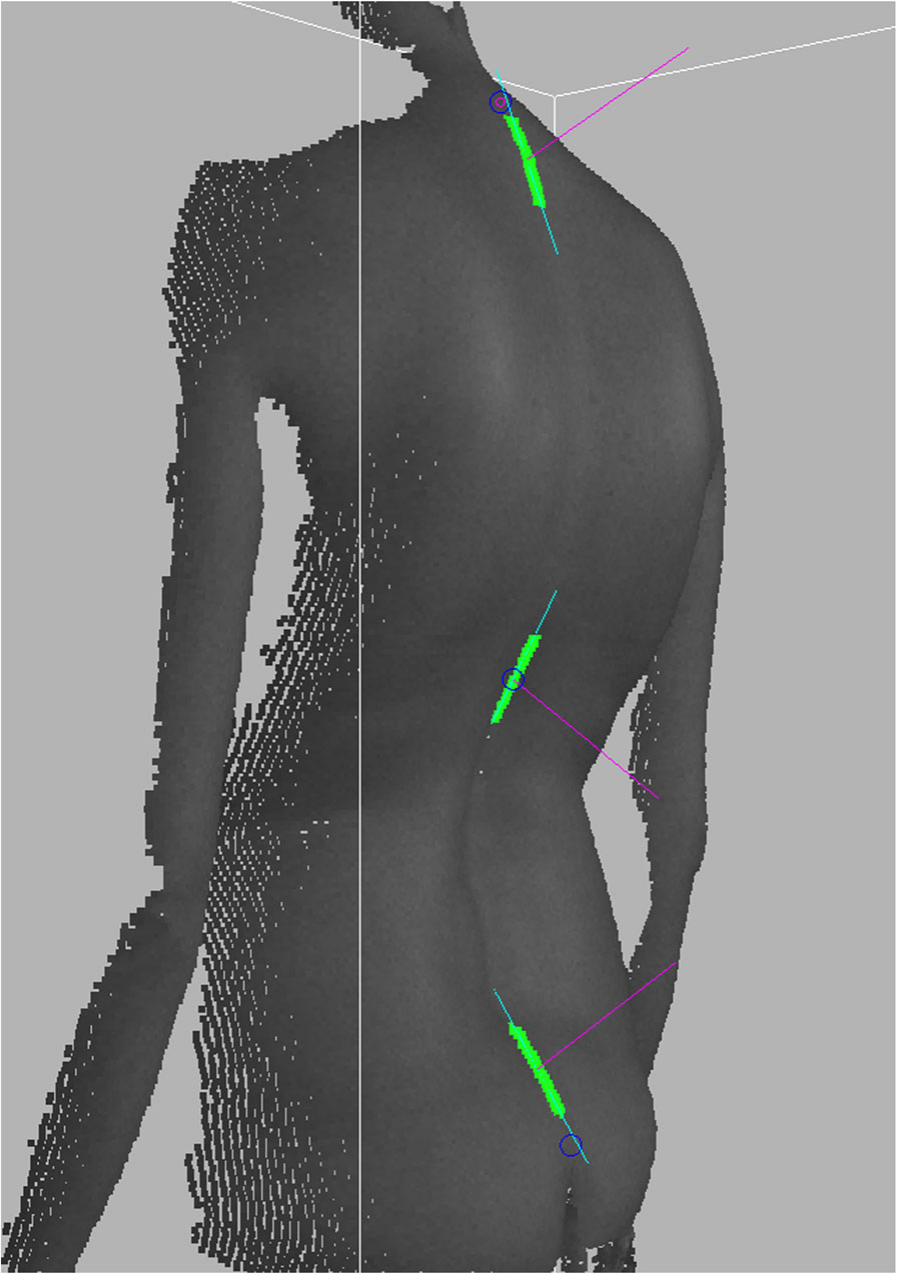
Areas selected in green were used for local best-fit planes (in *blue*, after projection on the sagittal plane). In magenta normal vectors used for angle calculation

The kyphosis and lordosis angles were estimated using an algorithm inspired by the Debrunner kyphometer [20]. Three rectangular areas of height 50 mm and width 10 mm, symmetrical with respect to the spine, were automatically found on the surface of the back at three levels of the spine (Fig. 2). The areas were to simulate the meeting points of the kyphometer with the surface of the back. The levels roughly corresponded to 20 mm below C7, 50 mm above intergluteal cleft and the transition point between lumbar lordosis and thoracic kyphosis. Points found in each area were used to calculate a best-fit plane. The kyphosis and lordosis angles were calculated as the angle between normal vectors of the top and middle, and middle and bottom planes, respectively. The normal vectors of the planes were first projected on the sagittal plane to obtain angles in this plane.

**Fig. 3:**
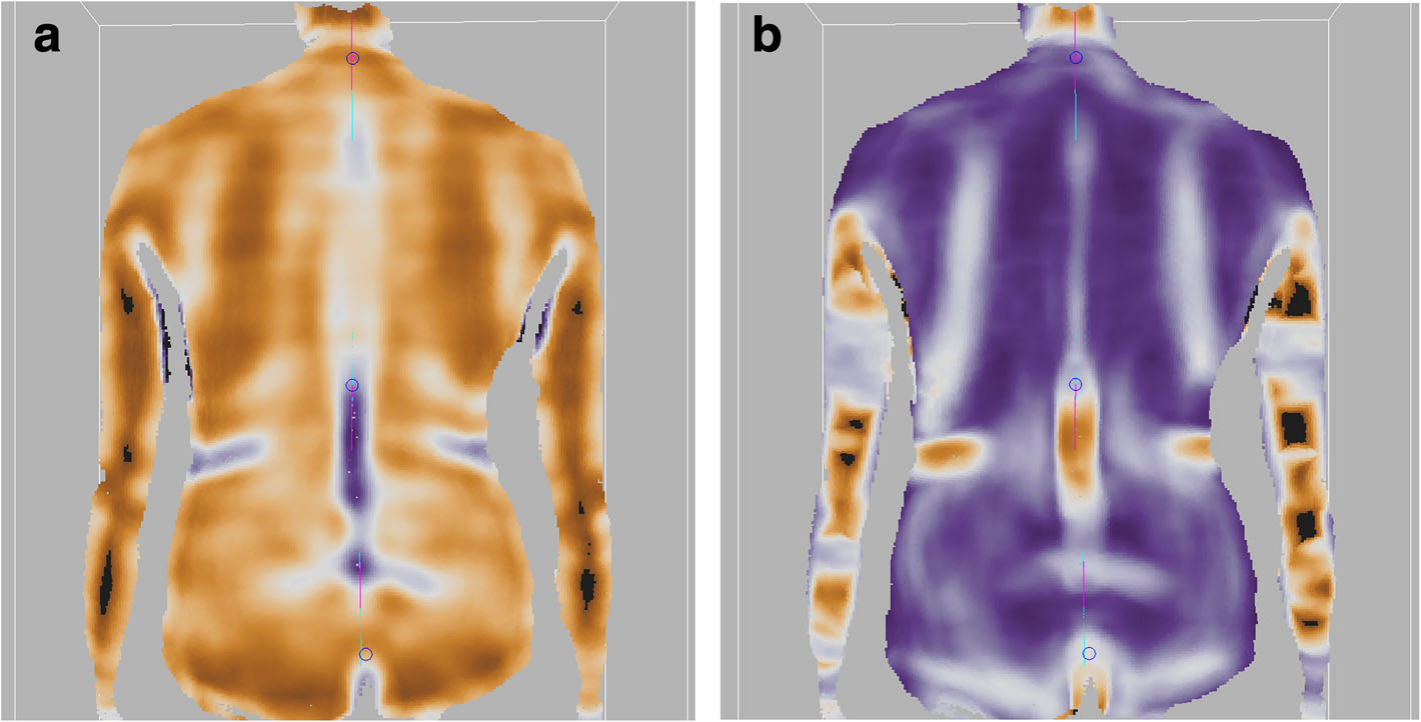
Curvature Index (**a**) and Normal Alignment Index (**b**) maps

The three levels were estimated using maps of Convexity Index (CI) and Normal Arrangement Index (NAI). The CI map allows to distinguish flat, concave and convex areas. The NAI map allows to distinguish cylindrical, plane and spherical areas. A detailed description of the algorithms used to calculate the maps is given in Appendix 1. The landmarks were chosen as the points with extreme values of CI (top and bottom areas) and zero value of NAI (middle) in the proximity of a point selected by the operator. Exemplary maps and automatically selected points are shown in Fig. 3.

**Fig. 4:**
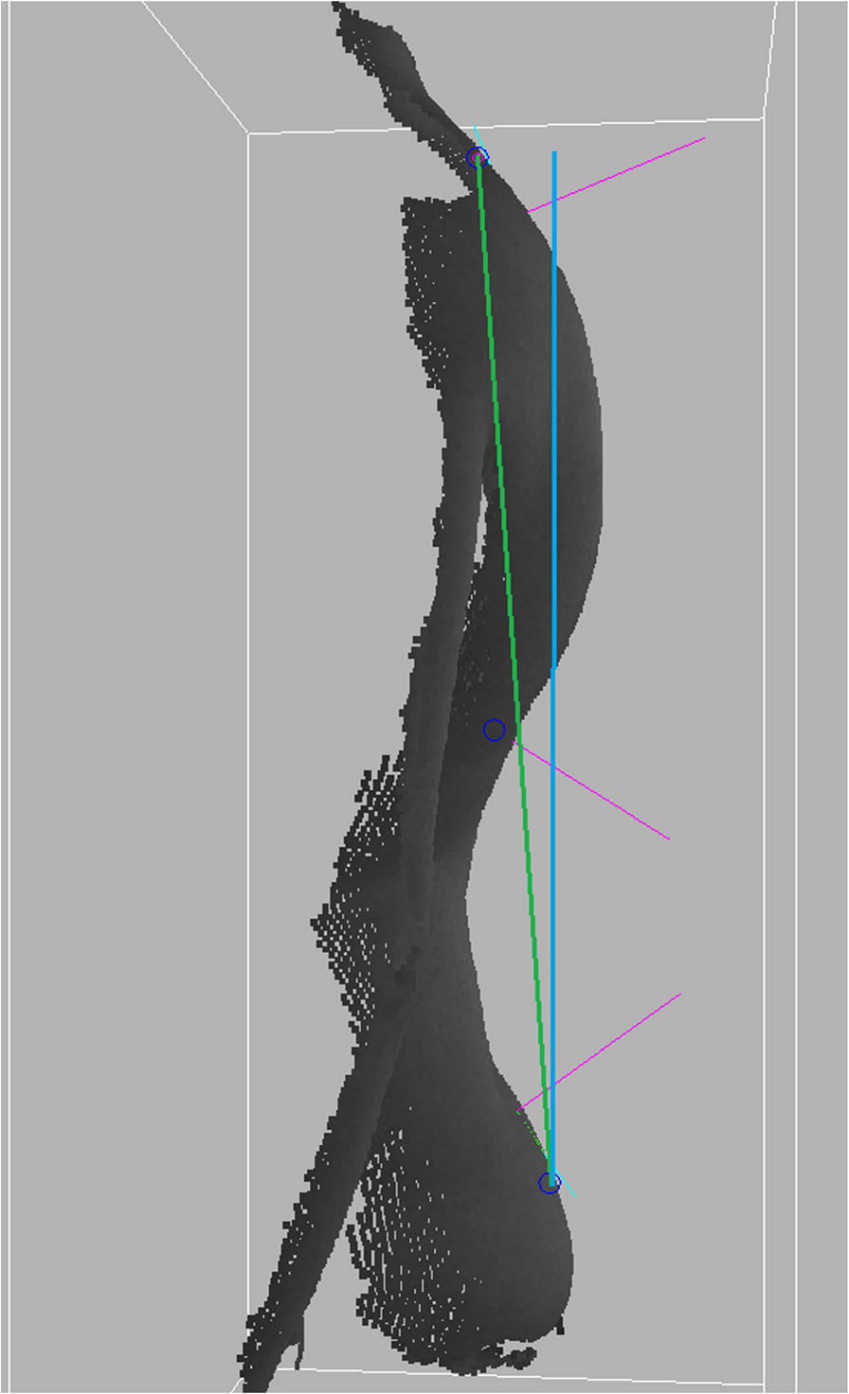
The figure shows the graphical result of the vertical balance angle measurement. The *green line* connects the bottom and top characteristic points. The *blue line* is the calibrated plumb line

The vertical balance angle was calculated as the angle between the line connecting the top and bottom characteristic points, and the plumb line (Fig. 4).

## Case presentation

**Fig. 5:**
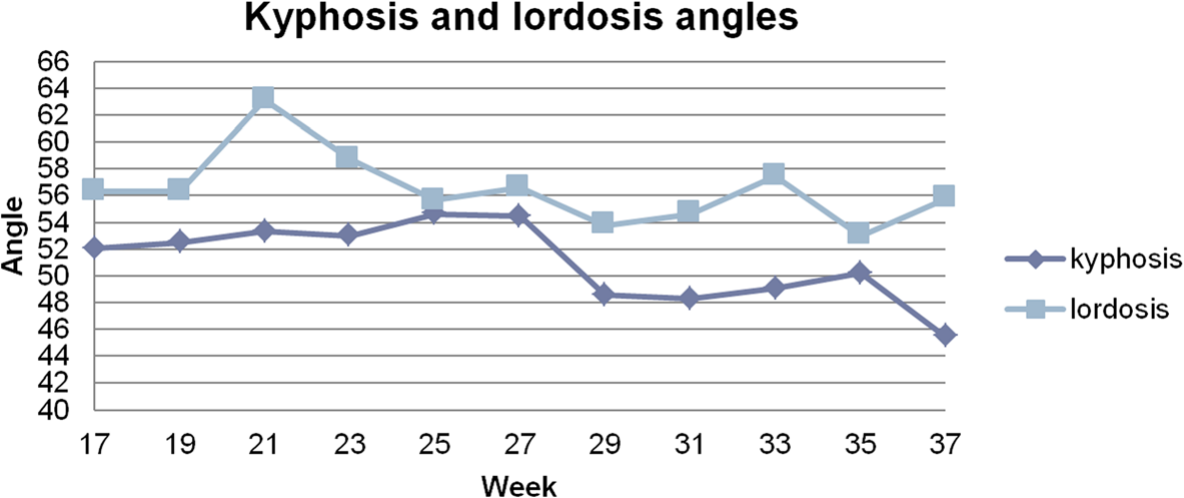
Median values of kyphosis and lordosis in each session

**Fig. 6:**
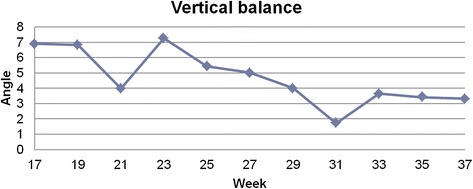
Median value of vertical balance angle in each session

**Fig. 7:**
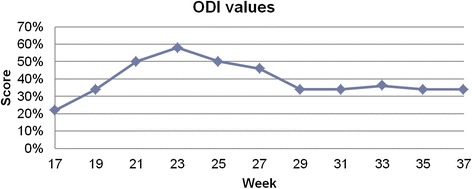
Values of the Oswestry Disability Index in each session

The values were correctly extracted from the measurement. In each session, three values were calculated, and the median value was chosen (only these values are presented). From these values mean and standard deviation were calculated. The results were: 50.9 ± 2.4° for kyphosis angle, 58.1 ± 2.1° for lordosis angle and 4.7 ± 1.7° for vertical balance angle. The registered change was 7.4° in kyphosis angle, 8.4° in lordosis angle and 5.5° in vertical balance angle (Figs 5 and 6). The calculated ODI values were between moderate disability and severe disability (22 to 58 %) (Fig. 7).

Interpretation is very limited due to lack of population data. However, we can observe that a major increase of lordosis angle in 21st week corresponds to a rapid decrease in vertical balance angle. In the 23rd week there is a maximum value of ODI and vertical balance angle, then vertical balance angle decreases along with the values of ODI, which would suggest a positive correlation between these two factors. After 27th week the kyphosis angle decreases along with the vertical balance angle with the lordosis angle being relatively stable, which would suggest straightening up of the subject. What is more after 33th week values of ODI and vertical balance are on the same level until 37^th^ week. The results obtained for the singular case cannot be generalized in any way. Nevertheless, they are consistent and plausible.

Measurements originating from surface topography systems are usually subject to validation with the gold standard–radiography. However, radiography should not be performed in pregnant women due to exposure to radiation, especially with such frequency. There are studies which recommend monitoring of scoliosis also with surface topography, regardless of the lack of direct correlation with the Cobb angle [21, 22]. The method presented in this study follows similar principles, and is intended for monitoring of changes in posture in time. Correlation with kyphosis and lordosis angles obtained from radiographs is desired, however not necessary in order to use the proposed method. Nevertheless, further research is required into validating the method on a larger group of women.

## Conclusions

This case study presents that surface topography may be suitable for monitoring of spinal curvature and posture change in pregnant women. The ionizing radiation studies are contraindicated during pregnancy. Surface topography data connected with information from pain level questionnaires allows to investigate the connection between changes in posture and back pain. Results of the study suggest that the period between the measurement sessions should not be much longer than 2 weeks. It is dictated by quite a high variability in data. Although, it remains unclear at the moment how much of this variability can be attributed to the method of the examination itself.

Using CI and NAI maps to find landmarks used in the calculation of curvature angles and extraction of vertical balance angle allows to obtain high reliability of such a study without the observer errors introduced otherwise.

The study produced plausible results, and we see potential in performing a randomized trial on a larger population. The main limitation of this study is the estimation of the levels where the thoracic kyphosis and lumbar lordosis angles were calculated. Although the characteristic points were computed automatically, the actual areas used for extraction of angles were chosen using a certain offset, chosen arbitrarily. We plan to address this problem in the future studies.

## Appendix 1

### Calculation of the Curvature Index and Normal Arrangement Index

The Curvature Index (CI) parameter describes how much the surface in the neighborhood of the considered point deviates from a plane. To calculate this value, points contained in the spherical neighborhood of a given radius are found, and a best-fit plane (BFP) is calculated. The BFP is a plane for which the sum of squared distances of all points to the plane is the lowest. Once they are obtained, the final value can be calculated:

1 $$ CI=\frac{{\displaystyle \sum_{i=1}^N\left({d}_i\cdot {w}_i\right)}}{{\displaystyle \sum_{i=1}^N{w}_i}} $$

where: *N*–a number of neighbors, *d*_*i*_–i-th neighbor distance to the BFP, *w*_*i*_–weight of i-th neighbor. The *w*_*i*_ value is related to the distance between the i-th neighbor and the considered point. The closer the neighbor to the considered point, the higher the weight is. In this way, neighbors that are closer to the considered point have a bigger influence on the curvature value. The *w*_*i*_ weight value is decreasing according to Gaussian function centered in the current point.

The Normal Arrangement Index (NAI) parameter describes the distribution of normal vectors in the neighborhood of the considered point, allowing to distinguish areas of unidirectional curvature (cylindrical), from areas of omnidirectional curvature (spherical).

All the points contained in the spherical neighborhood of a given radius are found to calculate the index. The value of NAI parameter is calculated with the use of another best-fit plane. If the values of the vectors are interpreted as points, the BFP^vect^ will be the plane best fit to these values. The final value is calculated as the mean distance of these points from BFP^vect^.

2 $$ NAI=\frac{{\displaystyle \sum_{i=1}^N\left|{d^{vect}}_i\right|}}{N} $$

where *d* ^*vect*^_*i*_–distance of the i-th normal vector’s end to the BFP^vect^, *N*–number of vectors (number of neighbors).
